# From Sand to Excellence: A Deep Dive into Abu Dhabi’s Rheumatology Landscape

**DOI:** 10.31138/mjr.011123.fst

**Published:** 2024-02-12

**Authors:** Khalid A. Alnaqbi, Fahad Fazal, Rajaie Namas

**Affiliations:** 1 Division of Rheumatology, Tawam Hospital, Al Ain, United Arab Emirates,; 2 Internal Medicine Department, College of Medicine & Health Sciences, United Arab Emirates,; 3 Rheumatology Department, Mediclinic, Al Ain, United Arab Emirates,; 4 Division of Rheumatology, Department of Internal Medicine, Cleveland Clinic Abu Dhabi, Abu Dhabi, United Arab Emirates

**Keywords:** Abu Dhabi, rheumatology, SEHA Company, PureHealth, medical tourism, research

## Abstract

The Emirate of Abu Dhabi (AD) is the capital and largest emirate of the United Arab Emirates (UAE). The emirate’s economic significance stems from non-oil and oil contributions to GDP. The 2022 GDP of Abu Dhabi was USD 230 billion. The government provides services to its residents through digital platforms such as official websites. The Abu Dhabi Health Insurance Law No. 23 of 2005 mandates that residents have access to necessary medical care and services. There is a paucity in the literature on the available rheumatology services in the Arab region. This review article aims to explore the status of rheumatology services in AD for both residents and visitors. It will include an overview of paediatric and adult rheumatology care, accessibility of diagnostic procedures, the integration of electronic medical records, access to medications, the status of postgraduate education, research, and suggestions on how to enhance rheumatology services in AD as a destination for medical tourism.

## INTRODUCTION TO THE EMIRATES OF ABU DHABI

The Emirate of Abu Dhabi (AD) is the capital and largest emirate of the United Arab Emirates (UAE). Its heterogenous population around mid-2016 was 2,908,173. The population growth rate of AD is 4.4%.^1^ The three main regions of the emirate are the city of AD, Al Ain, and Al Dhafra. The Gross Domestic Product (GDP) of AD for the year 2022 was AED 840 billion (USD 230 billion).^2^ The emirate’s economic significance stems from non-oil and oil contributions to GDP which constitute 52% and 48% at the 2022 prices respectively.^1^ Although the official language is Arabic, English is also used in most official communications including healthcare. AD government uses digital platforms, such as official websites, to distribute information about services to its residents.

There is a paucity in the literature on the available rheumatology care in the Arab region. This review article aims to discuss a detailed status of paediatric and adult rheumatology care and research, and suggest how to enhance rheumatology care in AD as a destination for medical tourism.

## DEPARTMENT OF HEALTH - HEALTH REGULATOR IN ABU DHABI

The Department of Health (DOH) is the regulatory body overseeing the health sector. It was established to ensure excellence in healthcare for the community by setting the strategy and policy for health services, ensuring they align with international standards.^3^ DOH offers a variety of electronic services via Tamm website.^4^ These include licensing for healthcare professionals and facilities, accrediting medical education and training centres, and listing authorised insurance providers and approved drugs.^5^

## MANDATORY HEALTH INSURANCE IN ABU DHABI

Implementing the mandatory health insurance scheme, known as the “Abu Dhabi Health Insurance Law,” Law No. 23 of 2005, ensures that individuals have access to necessary medical care and services, including rheumatology care.^6,7^ This inclusive approach reflects the emirate’s commitment to equitable healthcare and emphasises the emirate’s recognition of rheumatic diseases as a major public health concern.

### Insurance Companies

There are numerous health insurance providers in the UAE.^5^ The National Health Insurance Company, Daman, founded in AD in 2006 and owned by AD government, is the country’s leading health insurance entity. It makes use of digital accessibility via a smartphone application.^8^

## THE HEALTHCARE EVOLUTION IN ABU DHABI: SEHA, ADQ, AND MUBADALA HEALTH

Abu Dhabi Health Services Company (SEHA), established under Emiri Decree No. 10 of 2007, manages public health institutions in AD. SEHA manages more than 14 hospitals with over 3,000 beds, and over 46 clinics, employing over 14,000 people, including 2,900 physicians.^9^ “Seha” means “health” in Arabic. Among the facilities owned by SEHA Company are Sheikh Khalifa Medical City (SKMC), Sheikh Shakhbout Medical Centre (SSMC), Tawam Hospital, Al Ain Hospital, Sheikh Tahnoon Medical City, and Ambulatory Healthcare Services (AHS).

**Figure 1.**
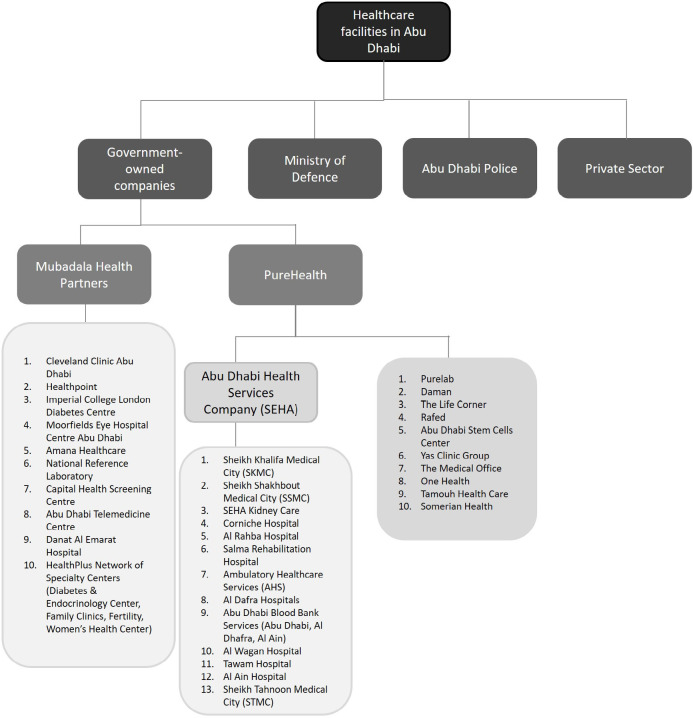
Healthcare facilities in Abu Dhabi.

Abu Dhabi Qabeda (ADQ) is an Abu Dhabi-based investment and holding company founded in 2018 to serve as a strategic partner of AD’s government.^10^ “Qadeba” means “holding” in Arabic. In January 2022, ADQ merged several companies under PureHealth, creating the UAE’s largest healthcare provider. This merger brought together entities such as SEHA Company, AHS, The Life Corner, Daman, and others (**Figure 1**). As a result, the scope of services expanded to include hospital management, laboratory services, medical supplies, and healthcare informatics.^11^

Mubadala Health is a subsidiary of Mubadala Investment Company, a forerunner in global investing. It aims to build a world-class healthcare infrastructure for the community by bridging together healthcare facilities and renowned international institutions. It oversees and manages specialised healthcare partners such as Cleveland Clinic Abu Dhabi (CCAD), Healthpoint, National Reference Laboratory, and Tawam Molecular imaging Centre.^12^

### Paediatric and Adult Rheumatology Care in Abu Dhabi

PureHealth (SEHA Company and Yas Clinic Group), Mubadala Health partners, and private healthcare facilities are the main healthcare providers in AD. Rheumatology services in AD are provided by approximately 43 adult rheumatologists and five paediatric rheumatologists. Notably, there are only three full-time academic rheumatologists at the College of Medicine and Health Sciences (CMHS), UAE University.

SEHA provides adult rheumatology services, and exclusively provides paediatric rheumatology care in AD.

In AD city and its neighbouring areas, Al Mafraq Hospital was the first to provide paediatric and adult rheumatology services. In November 2019, SEHA collaborated with the US-based non-profit Mayo Clinic, to jointly manage and operate SSMC.^13^ In 2020, Al Mafraq Hospital services were relocated to a newer hospital; Sheikh Shakhbout Medical City (SSMC). SKMC Hospital also provides both paediatric and adult rheumatology services. In May 2023, a joint ophthalmology–paediatric rheumatology Uveitis Clinic was established at SKMC.^14^

In Al Ain city and its surrounding areas, Tawam Hospital and Sheikh Tahnoon Medical City currently provide paediatric and adult rheumatology care. There are currently five adult subspecialty clinics managing spondyloarthritis, musculoskeletal ultrasound, systemic lupus erythematosus (SLE), interstitial lung disease (combining Rheumatology Respirology specialities), and Sjögren’s syndrome. Paediatric rheumatology services in Tawam Hospital are run by part-time consultants from the CMHS, UAE University. Al Ain Hospital provided adult rheumatology services from 2010 until August 2020, and provided paediatric rheumatology services from 2013 until August 2020 when it transitioned to a COVID-19 Centre.

Adult rheumatology clinics at AHS in Abu Dhabi and Al Ain are currently covered by rheumatologists both from SEHA and AHS. Furthermore, other providers, including CCAD, Healthpoint*,* Yas Clinic Group, Ministry of Defence, Abu Dhabi Police, and the private sector (e.g. Mediclinic) offer rheumatology services for adult patients.

## ELECTRONIC MEDICAL RECORDS (EMRs)

EMRs have transformed healthcare enabling organised, safe, and central storage of patient data, which improves patient care and collaboration among providers. They provide assistance with clinical documentation, test orders, referrals, and image viewing. The system may also include alerts and tools to improve patient safety and care outcomes. EMRs are now used in almost all hospitals in AD.

All healthcare facilities participate in the Malaffi Health Information Exchange (HIF) program, enabling the sharing of patient records across most healthcare facilities in AD.^15^ The plan to integrate with the EMR of Northern Emirates is currently underway and will offer physicians easy access to medical records, significantly reducing care costs and redundant tests.^16^

Examples of used EMRs in AD are Salamtak (Cerner^TM^) and Epic^TM^. Additionally, within the Salamtak system used in SEHA, rheumatologists have a dedicated platform integrating various rheumatology-related electronic patient-reported outcome measures (ePROMs) for both routine clinical practice and research.

## INVESTIGATIONS FOR PATIENTS WITH RHEUMATIC DISEASE

### Laboratory Tests

Almost all healthcare facilities that care for patients with rheumatic diseases provide basic laboratory tests for haematology, biochemistry, and serology for autoimmune rheumatic disease. Furthermore, more specialised tests are available in almost all the facilities, while some sophisticated tests are sent abroad. Some private laboratories provide genetic tests for various diseases, but they are not covered by insurance.

The National Reference Laboratory (NRL), a partner of Mubadala Health, was established in 2010. NRL operates a network of 10 facilities throughout the UAE and is staffed by over 20 pathologists and clinical scientists who specialise in various disease areas.^17^

In March 2023, Purelab Transplant Immunology Laboratory received the American Society Histocompatibility and Immunogenetics accreditation.^18^

### Imaging

Almost all major hospitals have access to a variety of imaging tests such as musculoskeletal (MSK) ultrasound, echocardiogram, MRI, MR angiography, CT angiography, dual-energy CT scan, whole-body bone scan, and bone densitometry. Some rheumatologists perform MSK ultrasound in their clinics, and some have obtained special certifications for this procedure. Positron Emission Tomography (PET)-CT scans are available in AD in the private sector, while it is available in Al Ain at Tawam Molecular Imaging Centre (under Mubadala Health). Nailfold capillaroscopy has seen limited use in rheumatology but is expected to become more widely used in the future.

### Tissue Biopsies

Tissue biopsies are an essential diagnostic and prognostic tool in patients with rheumatic diseases. Biopsies can be obtained from skin lesions, kidney, liver, lung, intestine, bone, blood vessels, muscles, nerves, and synovial membranes (though rarely performed). Biopsies can be performed by various specialties depending on the biopsy location and the expertise at the healthcare facility. Dermatology, gastroenterology, haematology, internal medicine, plastic surgery, orthopaedic surgery, and interventional radiology are among the available specialties.

Pathologists are accessible for accurate interpretation. Some hospitals have partnered with international centres and send certain biopsies (e.g. muscle or kidney) abroad for interpretation.

## MULTI-DISCIPLINARY SPECIALTIES SUPPORTING RHEUMATOLOGY

Due to the multi-systemic nature of rheumatic diseases, rheumatologists work closely with multi-disciplinary specialties which are available in most healthcare facilities in AD. This collaborative approach ensures accurate diagnoses, personalised treatment plans, and comprehensive management.

The first Psoriasis Preceptorship Program in the UAE was launched in Tawam Hospital from 27 to 28 September 2023. The program was attended by dermatologists from across the Gulf countries. It focused on the epidemiology, comorbidities, mimickers, outcome measures, and management of psoriasis and psoriatic arthritis internationally and in the Gulf region with an emphasis on the multidisciplinary team approach between dermatology and rheumatology.

There are no specialised rheumatology nurses in the UAE. SEHA is a role model for supporting nurses who work with rheumatologists. For example, after training nurses at Al Ain Hospital and allowing them to present at regional meetings on various nursing issues related to patients with rheumatic diseases, the first hands-on Rheumatology Nursing Preceptorship Program in the Gulf region was launched in December 2019 where rheumatologists and nurses provided hands-on experience for nurses from different hospitals in the UAE. Sessions included how to calculate various scores used for rheumatic patients, adherence, dealing with challenging patients, pregnancy, and vaccinations.

## AVAILABILITY AND ACCESSIBILITY OF RHEUMATIC DRUGS IN ABU DHABI

Most rheumatic medications including biologic disease modifying anti-rheumatic drugs, (DMARDs – both originators and biosimilars – as well as oral targeted synthetic DMARDs, are widely available and accessible in AD, as detailed in **Table 1**. Additionally, AD is among the first places globally to offer novel treatments for specific rheumatic diseases, especially once they are approved by international drug organisations like the US Food and Drug Administration (FDA) or the European Medicines Agency (EMA).^19^ This readily accessibility saves patients from the need to travel abroad for their treatment. Furthermore, if a rheumatologist at any healthcare centre in AD requires a medication that is not on the formulary list, the healthcare facility must submit a non-formulary medication request. Subsequently, the medication will be obtained.

**Table 1.** Availability of rheumatic medications in the Emirate of Abu Dhabi.

**Medications**	**Examples**
Disease modifying anti-rheumatic drugs (DMARDs)	
*Conventional synthetic*	methotrexate, hydroxychloroquine, sulfasalazine, leflunomide, azathioprine, mycophenolate mofetil, mycophenolic acid, cyclophosphamide, cyclosporine
*Targeted Synthetic*	apremilast, tofacitinib, baricitinib, and upadacitinib
*Biologic (originators and biosimilars)*	TNF inhibitor: etanercept [originator and biosimilars (Erelzi, Brenzys), adalimumab [originator Humira and biosimilars (Hyrimoz, Amjevita), infliximab [originator Remicade and biosimilars (Ixifi, Remsima), certolizumab, and SC golimumab Non-TNF inhibitors: tocilizumab, abatacept, rituximab [originator MabThera, and biosimilars (Rixathon, Ruxience), anakinra, canakinumab, ustekinumab, secukinumab, ixekizumab, belimumab (IV and SC), and anifrolumab
Anti-osteoporosis drugs	Bisphosphonates (alendronate, risedronate, zoledronate, pamidronate, ibrandronate), teriparatide, denosumab and romosozumab
Other medications	avacopan, nintadenib
Unregistered medications by pharmaceutical company and therefore are unavailable	abaloparatide, IV golimumab, sarilumab, rilonacept

IV: intravenous; SC; subcutaneous.

## ACCREDITATION OF RHEUMATOLOGY PROGRAMS IN ABU DHABI

The UAE achieved a significant milestone when Al Ain Hospital received rheumatology accreditation through the Joint Commission International (JCI) in April 2019. This recognition was for programs in Ankylosing spondylitis, SLE, rheumatoid arthritis, and Juvenile Idiopathic Arthritis, making it the first and only rheumatology centre outside the U.S. to achieve such prestigious accreditation.^20^ Obtaining this accreditation required several actions including updating the scope of services (adult and paediatric rheumatologists, nurses and rehabilitation), establishing structured infusion day care, developing a rheumatology nursing manual, and enhancing rheumatology interface within the EMR system. Furthermore, the development of evidence-based clinical practice guidelines, accompanied by key performance indicators (KPIs), ensured the delivery of high-quality structured programs. However, during the COVID-19 pandemic, rheumatology services were relocated to the nearby Tawam Hospital.

## MEDICAL TOURISM

Medical tourism involves individuals traveling from one country to another to access specific healthcare services. The travel and tourism sector contributed nearly AED 167 billion to the UAE’s GDP in 2022, accounting for 9% of the total GDP. The UAE holds the 25^th^ position in the global Travel and Tourism Development Index.^21^

Abu Dhabi’s focus on medical tourism has resulted in partnerships with prestigious healthcare institutions around the world, further elevating its reputation as a centre for advanced medical treatment and wellness services. When seeking a medical opinion, important factors such as accreditation, the physician’s qualifications, travel logistics, and insurance coverage must be considered. In line with the Jawda (meaning “quality” in Arabic) Quality framework program and Abu Dhabi’s Vision 2030, the emirate collaborates with experts across tourism, transportation, immigration, and healthcare, aiming to provide highly specialised healthcare to international patients. To achieve this, several measures have been implemented. Many healthcare facilities in AD have received international accreditation, most notably from the JCI, ensuring that they meet global healthcare quality and patient safety standards. Furthermore, many rheumatologists in AD have completed international training programs and bring distinct clinical and research backgrounds to their practice.

The ease of obtaining visa, combined with an advanced aviation infrastructure and a variety of payment options—including international insurance or self-pay—makes AD a popular destination for patients seeking medical care. DOH, in collaboration with the Department of Culture and Tourism – Abu Dhabi, launched the Abu Dhabi Medical Tourism e-portal. This digital platform provides visitors with comprehensive information on the health-care services available during their stay. It highlights over 287 medical treatments provided by more than 173 physicians across the emirate, and features 40 health-care facilities adhering to the DOH’s Jawda program standards. The Jawda program’s quality metrics assist the DOH in selecting facilities for the medical tourism network, ensuring they maintain high standards and offer unique medical services in comparison to other healthcare models.

Additionally, the DOH grants accreditation to centres of excellence (CoE) in the UAE that meet specific criteria. While the UAE does not currently have a designated Arthritis CoE, other CoEs in AD include the Haematopoietic Stem Cell Transplant, Stroke, and Adult Cardiac Surgery CoEs.^22^

## INVESTMENT IN POST-GRADUATE RHEUMATOLOGY TRAINING

Emiratisation is a strategic initiative by the UAE government to increase the number of Emiratis in the public and private sectors. The primary goal is to integrate UAE nationals into the country’s workforce, ensuring their active participation in the economic development of the nation.

Numerous measures have been implemented to integrate Emiratis into medical specialties, like rheumatology, and leadership positions. SEHA serves as a prime example of such an endeavour. To ensure the availability of local experts who understand the UAE culture, additional rheumatology specialisation, either abroad or locally, is offered.

### Global Approach to Rheumatology Training for Emirati Physicians

Before national rheumatology fellowship programs were established, AD sponsored Emirati physicians to pursue rheumatology specialisations in countries like Canada, the USA, Ireland, Germany, and France. This approach allowed them to gain a wide range of clinical experiences. There are currently four Emirati rheumatologists who completed subspecialty training abroad in spondyloarthritis, musculoskeletal ultrasonography and SLE.

### National Rheumatology Fellowship Programs

The first adult and paediatric rheumatology fellowship programs in the UAE were established at SSMC in October 2019, and September 2022 respectively. The curricula were adopted and were accredited by the Accreditation Council for Graduate Medical Education international (ACGME-i). Each program has a duration of three years.

The National Institute for Health Specialties (NIHS) formed the Scientific Committee of the Paediatric and Adult Rheumatology Fellowship programs on 12 October 2021. The committee was made up of 12 members from various healthcare institutions across the country (9 adult rheumatologists, and 3 paediatric rheumatologists). It held a number of meetings to develop the Paediatric and Adult Rheumatology fellowship curricula, logbooks, milestones, program information forms, rubrics, and entrustable professional activities. The ACGME and the Royal College of Physicians and Surgeons of Canada were used as models. Each fellowship program lasts 3 years. The NIHS approved both programs in 2022.^23^

Graduates of the SSMC adult fellowship program can sit for the Jordanian Board of Rheumatology. Furthermore, the Arab Board of Health Specialisation established the Arab Board of Adult Rheumatology fellowship program in June 2023, providing another certification opportunity for graduating fellows.^24^ In November 2023, NIHS awarded accreditation to the paediatric fellowship program at SSMC, followed by the accreditation for the adult fellowship program in January 2024. In collaboration with the Saudi Central Board for Accreditation of Health Care Institutions (CBAHI), efforts are underway to develop the exit examination, which will grant physicians the Emirati Board of Rheumatology certification upon passing.

## RHEUMATOLOGY CARE DURING COVID-19 PANDEMIC

During the COVID-19 crisis, rheumatology services throughout the world faced significant challenges and adaptations. Many in-person consultations transitioned to telemedicine platforms to ensure patient safety while maintaining continuity of care. The management of immunosuppressed patients required special consideration given their potential increased vulnerability to the virus.^25^ Telemedicine visits across healthcare facilities in AD, which have been employed since the start of the COVID-19 pandemic in 2020, have transformed the care of rheumatic diseases. This method is especially useful for patients with rheumatic conditions like gout or osteoporosis, where in-person visits may be unnecessary, or when discussing less critical test results. Telemedicine in some hospitals, such as Mediclinic and CCAD, involves patients logging into an application with a camera on their smartphones and communicating directly with their caregivers.

Furthermore, many hospitals in the UAE, including those in AD, started providing free medication delivery to patients in order to improve their adherence. This service continues to be available as needed.

### Rheumatology Initiatives of SEHA during COVID-19 Crisis

During the early months of COVID-19 pandemic, Al Ain Hospital was the first in the Emirate of AD to exclusively serve COVID-19 patients. As a result, many services were relocated to the nearby Tawam Hospital.

The rheumatology leadership at SEHA took proactive measures to adapt their patient care. They contributed to updating the National Guidelines for Clinical Management Treatment of COVID-19, especially when tocilizumab was prescribed as empirical therapy for COVID-19-related cytokine storm syndrome.

The rheumatology leadership developed clinical practice guidelines to streamline rheumatology care throughout the pandemic. These guidelines encompassed tele-consultation procedures, adjustments to infusion day care, and on-call rheumatology services for admitted patients experiencing COVID-19-related cytokine storm syndrome. Furthermore, SEHA hospitals and AHS facilitated laboratory testing for rheumatic patients, reducing the need for them to visit SEHA hospitals directly. Additionally, rheumatic patients stable on intravenous therapies, such as abatacept, tocilizumab, and belimumab, were recommended to switch to subcutaneous administration of these biologics. In addition to these clinical adjustments, the leadership was also involved with the Arab Adult Arthritis Awareness (AAAA) Group in the development and distribution of educational pamphlets for patients. These materials provided the latest information available at that time regarding rheumatic medications during the pandemic.^26^

## RHEUMATOLOGY RESEARCH IN ABU DHABI

Some healthcare facilities provide eLibrary to serve as a useful resource for researchers. For example, SEHA eLibrary provides online webinars to guide researchers with various issues, particularly addressing how to write a paper and master the role of an effective peer reviewer. It also offers evidence-based medical research from international publications, randomised controlled trials, systematic reviews, multimedia-based interventions, and drug databases, among others. It includes 13 databases, over 2,400 high-impact factor journals with full-text access, 2,000 subscribed journals, around 20,000 Medline Complete journals, and 2,000 books.^27^ UpToDate and DynaMed are also integrated into the EMR (Salamtak) system, to aid in point-of-care decision-making.

### Regional Research

In 1993, the first paper in rheumatology in the UAE about rheumatoid arthritis cohort from AD was published.^28^ In 1995, the first paper in the Gulf region about the prevalence of HLA-B27 in healthy adults in AD was published.^29^ Paediatric and adult rheumatologists from healthcare facilities in AD have contributed to the literature with original research, review papers, editorials and case reports on a range of rheumatic diseases, including ankylosing spondylitis, SLE, reactive arthritis, anti-phospholipid antibody syndrome, juvenile idiopathic arthritis, uveitis, and neonatal lupus.^30–37^

The first landmark paper on the UAE scleroderma registry incorporated data from both government and private hospitals across the UAE including CCAD, SKMC, SSMC and Tawam Hospital.^38^ Furthermore, rheumatologists from Tawam Hospital and CCAD participated in the first consensus statements on non-pharmacological and pharmacological management of psoriatic arthritis.^39,40^ More consensus recommendations on various rheumatic diseases are currently underway.^41,42^

### International Research

Adult rheumatologists in AD have contributed to and published several international studies. For example, SEHA rheumatologists participated in the 2021 recommendations on telemedicine in rheumatology developed by both the Asia Pacific League of Associations for Rheumatology (APLAR) and the Arab League of Association for Rheumatology (ArLAR),^43, 44^ the adaptation of the 2015 American College of Rheumatology (ACR) treatment guideline for rheumatoid arthritis for the Eastern Mediterranean region.^45,46^ Al Ain Hospital participated in international multi-centre studies such as PROOF study on axial spondyloarthritis.^47^ Additionally, rheumatologists at Tawam Hospital have collaborated with an international task force and published the comparative analysis and creation of a roadmap for sustainable biosimilar markets.^48^

Furthermore, paediatric rheumatologists in AD have contributed to numerous regional and international studies on paediatric cohorts with rheumatic diseases such as juvenile idiopathic arthritis, systemic autoinflammatory diseases, and uveitis.^49–53^

Numerous research abstracts in paediatric and adult rheumatology of AD have been presented at national, regional (e.g. Egypt, Saudi Arabia, and Kuwait), and international conferences (e.g. Korea, Italy, Spain, Morocco, and USA).^41,54–60^

## FUTURE DIRECTIONS IN RHEUMATOLOGY CARE IN ABU DHABI

We present here some suggestions for improving rheumatology care in AD, with the goal of making it a leading hub for both rheumatology care and research.

### Enriching Comprehensive Rheumatology Care with Multi-disciplinary Specialists

Immunologists (paediatric and adult), sports medicine physicians and MSK radiologists are essential members of the multi-disciplinary team who provide comprehensive rheumatology care. Currently, the number of physicians specialising in these areas in the UAE is limited. Immunologists provide expert opinions to rheumatologists on complex cases, as well as for those primarily related to immunological and allergic diseases. Furthermore, there are many referrals to rheumatologists for sports-related injuries. MSK radiologists play an important role in diagnosing paediatric and adult patients with various rheumatic, orthopaedic, or sports medicine conditions.

### Role Diversification and Subspecialisation in Rheumatology

Encouraging subspecialty training in specific areas of rheumatology will ensure local expertise for referrals. Furthermore, recognising and formalising the role of rheumatology nurses can greatly enhance patient care. Such initiatives will help establish more rheumatology subspecialty clinics and CoEs.

### Utilisation of ePROMs for Quality Assurance

Integrating ePROMs into the EMR systems will allow rheumatologists to include these instruments in their daily practice and track their usage through KPIs. This will ensure the provision of a consistently high-quality service.

### Tailoring Treatment: Personalised Medicine

Personalised medicine is gaining attraction in patient care. By ensuring the availability of genetic and molecular tests locally, rheumatologists can tailor their management to a patient’s unique genetic makeup, lifestyle, and environmental factors. Expanding local testing capabilities will minimise outsourcing to laboratories abroad. This could potentially enhance the effectiveness and safety of medical care, minimise adverse reactions to treatments, and improve patient outcomes.

### Strengthening the Rheumatology Research Infrastructure

There is a growing need for a robust infrastructure dedicated to advancing rheumatology research, and achieving this goal entails a number of critical steps. Firstly, it necessitates protected research time for rheumatologists with a proven track record of successful research. Secondly, it requires establishing a dedicated research office, and appointing research coordinators. Thirdly, web-based platforms will play a critical role in this transformation by facilitating the creation of databases or registries of various rheumatic diseases, ensuring all patient data is accessible and organised. The success of the UAE Scleroderma registry serves as a motivation for establishing more rheumatic disease registries. Fourthly, having access to a biostatistician is essential. Lastly, ensuring the availability of (preferably centralised) eLibraries across major healthcare facilities will pave the road towards enhancing research infrastructure.

Unfortunately, many international multi-centre randomised controlled trials, especially those assessing medication efficacy, exclude Arab countries. Because of this exclusion, drug efficacies observed elsewhere might not necessarily reflect similar outcomes in our region. Hence, launching electronic registries in AD has the potential to significantly transform the landscape of rheumatology research and care in the region.

By strengthening this research infrastructure, AD will continue providing significant contributions to the rheumatology field while also positioning itself as an attractive destination for international pharmaceutical companies looking to conduct clinical trials.

### Strengthening Global Rheumatology Connections

AD can further elevate its clinical and research standards by forming alliances with renowned international rheumatology centres and research institutions. Such collaborations promise mutual knowledge exchange, joint clinical trials, and the adoption of best practices from around the world.

### Promoting Patient-Centric Care

Establishing a Patient Support Group is essential for spreading awareness and ensuring that patients’ needs are consistently met, and their concerns are addressed. Additionally, many consensus recommendations require the involvement of patient representatives.^41,44^

### Raising Public Awareness of Rheumatic Diseases

Sporadic public awareness campaigns on various rheumatic diseases, such as ankylosing spondylitis, psoriatic arthritis, SLE and osteoporosis have helped emphasise the importance of early diagnosis and intervention. Most of these initiatives took place within the premises of the organising hospitals, in shopping malls, or at government offices such as municipalities. Healthcare institutions would benefit from aligning their agendas with established World Health Days, such as World Arthritis Day and World Ankylosing Spondylitis Day. Engaging stakeholders from different administrations would greatly support efforts to raise public awareness.

## CONCLUSION

Healthcare facilities in AD demonstrate a commitment to excellence in rheumatology care. Rheumatologists can accurately diagnose and monitor rheumatic disease by utilising the latest technology resulting in timely treatments and better patient outcomes. The availability and accessibility of the latest rheumatic medications and multidisciplinary specialties deliver comprehensive care tailored to each rheumatic patient’s needs. Collaborations with prestigious global medical institutions, adherence to international accreditation standards such as the JCI, and advancement of medical training and research help establish AD as a destination for medical tourism.

## ABBREVIATIONS

ACGMEi: Accreditation Council for Graduate Medical Education international
AD: Abu Dhabi
ADQ: Abu Dhabi Qabeda
AHS: Ambulatory Healthcare Services
APLAR: Asia Pacific League of Associations for Rheumatology
ArLAR: Arab League of Association for Rheumatology
CBAHI: Saudi Central Board for Accreditation of Healthcare Institutions
CCAD: Cleveland Clinic Abu Dhabi
CMHS: College of Medicine and Health Sciences
CoE: Centre of Excellence
DMARDs: Disease-Modifying Anti-Rheumatic Drugs
DOH: Department of Health
EMR: Electronic Medical Records
ePROMs: electronic Patient-Reported Outcome Measures
GPD: Gross Domestic Product
HIF: Health Information Exchange
JCI: Joint Commission International
KPIs: Key Performance Indicators
MSK: musculoskeletal
NIHS: National Institute for Health Specialties
NRL: National Reference Laboratory
PET: Positron Emission Tomography
SKMC: Sheikh Khalifa Medical City
SLE: Systemic Lupus Erythematosus
SSMC: Sheikh Shakhbout Medical City
UAE: United Arab Emirates
